# Association Between Anti-bacterial Drug Use and Digestive System Neoplasms: A Systematic Review and Meta-analysis

**DOI:** 10.3389/fonc.2019.01298

**Published:** 2019-11-27

**Authors:** Chongxi Bao, Ke Wang, Yudi Ding, Jinliang Kong

**Affiliations:** Department of Respiratory Disease, First Affiliated Hospital of Guangxi Medical University, Nanning, China

**Keywords:** anti-bacterial drugs, cancers, risk, systematic review, meta-analysis

## Abstract

**Background:** Anti-bacterial drugs are thought to be associated with several malignancies.

**Objective:** We conducted a systematic review and meta-analysis to assess the association between antibacterial drug exposure and the risk of digestive system neoplasms.

**Methods:** Relevant publications reporting a relationship between antibiotic use and the risk of cancer were identified in PubMed, EMBASE, and Cochrane Central Register through June 2018. The random-effects model was selected to pool the risk ratios (RRs) and determine 95% confidence intervals (95% CIs). We performed subgroup analyses by tumor organ site, individual antibacterial drug class, and drug dose accumulation.

**Results:** A total of 17 eligible studies (four randomized trials and 13 observational studies) involving 77,284 cancer patients were included in our analyses. Anti-bacterial drug exposure slightly increased the risk of overall digestive system cancer (RR, 1.12; 95% CI, 1.10–1.14), stomach and small intestine (RR, 1.12; 95% CI, 1.07–1.17), anorectocolonic (RR, 1.08; 95% CI, 1.05–1.12), and hepatobiliary and pancreatic cancers (RR, 1.18; 95% CI, 1.14–1.22). For different anti-bacterial drugs classes, nitroimidazoles (RR, 1.17; 95% CI, 1.09–1.26) and quinolones (RR, 1.18; 95% CI, 1.11–1.26) showed a modest association with the risk of cancers incidence. The risks of digestive system cancers increased with the rise of drug dose accumulation: low (RR, 1.08; 95% CI, 1.05–1.11), intermediate (RR, 1.15; 95% CI, 1.12–1.18), and high (RR, 1.22; 95% CI, 1.18–1.26).

**Conclusions:** Anti-bacterial drug exposure was associated with the risks of digestive system cancer occurrence in our analysis.

## Introduction

Digestive system cancers, such as esophageal, stomach and small intestine, anorectocolonic, hepatobiliary, and pancreatic cancers, are a global threat to human health and a leading cause of cancer death (1–3). The identification of etiological factors is important to preventing the occurrence of these cancers and decreasing death rates. Relevant publications have suggested the hypothesis that the use of certain drugs is associated with cancer development (4, 5), and that the regular use of anti-bacterial drugs is involved (6). The risk of several malignancies, such as lung (7), hematologic (8), and breast cancer (9), are reportedly associated with anti-bacterial drug exposure. The risk of colorectal and several other digestive cancers have also been evaluated in previous studies (10, 11). To our knowledge, there have not been any systematic reviews or meta-analyses examining the association between digestive system cancers and antibacterial drugs.

With the concern of the global, widespread use of anti-bacterial agents, the effects on drug-induced cancer create uncertainty. We conducted a systematic review and meta-analysis to assess the association between the risk of digestive neoplasms and anti-bacterial drug exposure.

## Methods

We performed the systematic review based on the Preferred Reporting Items for Systematic Reviews and Meta-analyses guidelines (12). The protocol for our meta-analysis was documented online (PROSPERO registry- CRD42018098646).

### Data Sources and Search

A search of the relevant publications (in English) up to June 2018 was conducted in PubMed, EMBASE, and the Cochrane central register. Case-control, cohort studies, and randomized control trials (RCT) were identified by searching with the following Medical Subject Heading (Mesh) terms and text words: “neoplasms,” “tumors,” “tumor,” “cancer,” “cancers,” “malignant neoplasms,” “neoplasm,” and “anti-bacterial agents,” “anti-bacterial compounds,” “anti-infective agents,” “anti-mycobacterial agents,” and “risk.” Additional literature was searched by scanning the reference lists of included studies. Abstracts and titles were reviewed in the primary search independently by both investigators (Bao Chongxi, Ding Yudi). Potentially relevant articles were evaluated in detail, and disagreements between investigators were resolved by consensus. Studies were included if they met the following criteria: (1) Study reported the association between antibiotic exposure and the digestive system cancer risk; (2) Study provided four-fold table data or effect sizes, such as risk ratio (RR), relative risk (RR), and odds ratio (OR); (3) Study used prospective or retrospective, cohort or (nested) case-control designs. When multiple articles originated from the same population, we would combine these articles into one study, and used the most applicable data, or data with the longest follow-up duration.

### Data Abstraction

Two investigators extracted all data independently (Chongxi Bao, Yudi Ding). Data was double checked for accuracy after finishing the individual work, and arguments were resolved by discussion. Information needed for the meta-analysis that was absent from any of the selected studies was obtained by contacting the authors via email. The following data were extracted from each included study: journal name, first author name, publication year, country or region, study type (case–control studies/cohort studies/nested case–control studies/RCTs), follow-up period, total patients, number of anti-bacterial drugs exposed to (which could include 0), detailed prescription of anti-bacterial drug use, RRs, ORs, or SIRs, and the corresponding 95% confidence intervals (CIs).

### Quality Assessment

The two investigators (Ke Wang, Jinliang Kong) used the Newcastle-Ottawa Scale (NOS) to assess the quality of cohort or nested case-control studies independently (13), and a study of high quality was defined as one with seven or more stars (13, 14). The quality of the included RCTs was evaluated using the Cochrane risk of bias tool (15).

### Statistical Analysis

All data analyses were performed using the COMPREHENSIVE META-ANALYSIS software version 3.0 (Biostat, Englewood, NJ). A significant degree of heterogeneity between studies was defined using the *I*^2^ statistic value (16, 17), and the random effects model was selected according to heterogeneity (*p* < 0.1, *I*^2^ > 50%) (17). Publication bias was examined by Egger's regression asymmetry test and funnel plot (18, 19). Given that digestive cancers occur rarely in the general population, the distortions of various effect sizes (RR, OR, SIR) were ignored, and we used RR as the general effect size (20, 21).

We performed subgroup analyses according to different digestive system organ sites involving the esophagus, stomach and small intestine, anorectocolonic, hepatobiliary system, and pancreas (9, 10). Risks of digestive system cancers associated with individual anti-bacterial drug exposure were also assessed. The potential effect of drug dose accumulation on tumor development was evaluated by the subgroups: low, intermediate, and high cumulative prescription doses (22). Drug dose cumulative grade was based on the percentile of prescriptions or treatment courses within users: low (prescriptions/courses < 2), intermediate (prescriptions/courses 2–5), and high (prescriptions/courses > 5) (10, 23). Considering that cancer development is a step-wise process occurring over a period of several years, we conducted subgroup analyses by follow-up period before cancer diagnosis based on 25 and 50% of the duration (years). RR (95% CIs) for subgroups of tumor sites, categorical anti-bacterial drugs, and drug dose cumulative grades were calculated.

## Results

### Included Studies and Study Characteristics

**Figure 1:**
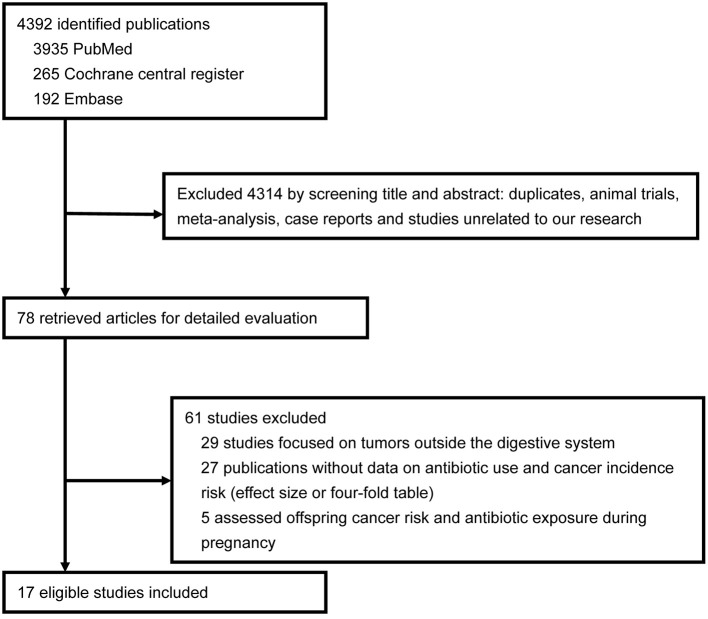
Flowsheet of literature search and study selection.

A total of 4,392 articles were initially identified from the search in the selected databases and 78 articles were retrieved and further reviewed after the screening of titles and abstracts. We ultimately identified 17 eligible studies including 13 observational studies (6, 9–11, 23–31) (cohort and nested case-control) and four RCTs (32–35) (Figure 1). The characteristics of the included studies are summarized in Table 1. These studies included 77,284 cancer patients from 18,205,771 individuals. Anti-bacterial drugs were used to eradicate helicobacter pylori (Hp) in seven studies (29–35), and two study cohorts consisted of patients with peptic ulcer disease. Detailed anti-bacterial agent classifications were not provided in seven of the above studies, and we classified another four studies (9, 24, 25, 28) as unknown with regard to anti-bacterial drug class subgroup. For the risk evaluation outcome data, the number of cancer patients with anti-bacterial drug exposure (or not) was shown in 11 studies (Supplementary Table 1), RR (95% CIs) was shown in three studies, SIR (95% CIs) was shown in two studies, and OR (95% CIs) was shown in one study. Drug dose accumulation data (prescriptions or treatment courses) was obtained from six studies (10, 11, 23, 25, 27, 28). All of the included studies excluded patients with only a 1–2 years interval before cancer diagnosis because the cancers were unlikely to develop in such a short time.

**Table 1:** Summary of included study characteristics.

**References**	**Country**	**Design**	**Follow-up (years)**	**Total cancer cases (*n*)**	**Cohort (*n*)**	**Data format**	**Anti-bacterial drugs classification^c^**	**Tumor Sites**
Fall et al. (24)	Sweden	Cohort	11.8	645	501,757	SIR	All anti-bacterial drug	Gastric
Kilkkinen et al. (25)	Finland	Cohort	7	26,373	3,112,624	RR	All anti-bacterial drug	Esophagus, gastric, duodenum, colon, rectum, liver, gall bladder, pancreas
Take et al. (29)	Japan	Cohort	3.9	13	1,342	Events number	Hp eradication in patients with peptic ulcer disease	Gastric
Lee et al. (30)	China	Cohort	8	31	4,121	Events number	Hp eradication	Gastric
Wu et al. (31)	China	Cohort	5.92–7.22^a^	249	80,255	SIR	Hp eradication in patients with peptic ulcer disease	Gastric
Didham et al. (9)	New Zealand	Nested case–control	4.5^b^	1,189	6,500	OR	Penicillins, macrolides, cephalosporins, sulphonamides, tetracycline, nitrofurantoin	Esophagus, stomach and small intestine, colorectal, liver, pancreas and other digestive
Friedman et al. (6)	USA	Nested case–control	12.5	79	6,608,681	RR	Azithromycin, ciprofloxacin, clarithromycin, sulfamethoxazole	Anus, anal canal, anorectum
Friedman et al. (26)	USA	Nested case–control	12	149	6,500,000	RR	Metronidazole	Liver, intrahepatic bile ducts, colon, anus, anacanal, anorectum
Wang et al. (28)	China	Nested case–control	7.4	5,572	640,173	Events number	Anti-aerobic and anti-anaerobic agents	Colon, rectal
Boursi et al. (10)	UK	Nested case–control	6-7	16,654	615,951	Events number	Penicillins, macrolides, cephalosporins, sulfacetamides, tetracyclines, quinolones, nitroimidazoles	Esophagus, gastric, hepatocellular, biliary, gallbladder, pancreas
Boursi et al. (27)	UK	Nested case–control	6.5	20,990	103,044	Events number	Penicillins, macrolides, cephalosporins, sulfacetamides, tetracyclines, quinolones, nitroimidazoles	Colorectal
Dik et al. (23)	Netherlands	Nested case–control	5	4,029	20,017	Events number	Penicillin, tetracyclines, sulphonamides, macrolides, quinolones, nitrofurans	Colorectal
Yang et al. (11)	UK	Nested case–control	11	1,195	5,835	Events number	All anti-bacterial drugs	Primary liver cancer
Correa et al. (32)	Colombia	RCT	6	5	1,219	Events number	Hp eradication	Gastric
Wong et al. (34)	China	RCT	8	18	988	Events number	Hp eradication	Gastric
Li et al. (33)	China	RCT	15	84	2,258	Events number	Hp eradication	Gastric
Zhou et al. (35)	China	RCT	10	9	1,006	Events number	Hp eradication	Gastric

*OR, odds ratio; RR, risk ratio; RCT, randomized controlled trial; SIR: standardized incidence ratio; Hp, helicobacter pylori*.

a *Mean follow-up period for early Hp eradication population was 5.92 years, and the later eradication was 7.22 years*.

b *Follow-up period range from 2 to 7 years, we used the median 4.5 years*.

c *“Hp eradication” indicates anti-bacterial drugs classes used to treat Hp infection*.

### Study Quality, Publication Bias, and Heterogeneity

Thirteen observational studies were of significant high quality (scores ranged from 7 to 9) and four RCTs were assessed as moderate or high quality (Supplementary Tables 2, 3). Egger's test (*p* = 0.68) and funnel plot (Supplementary Figure 1) did not indicate obvious publication bias. We conducted sensitivity analyses after excluding individual studies, and no excessive differences in summary outcomes were observed. The random effects model was selected to pool the RR (95% CIs) for statistically significant heterogeneity (*p* < 0.01, *I*^2^ = 77%).

### Overall Anti-bacterial Drug Exposure and Digestive System Cancer Risk

**Figure 2:**
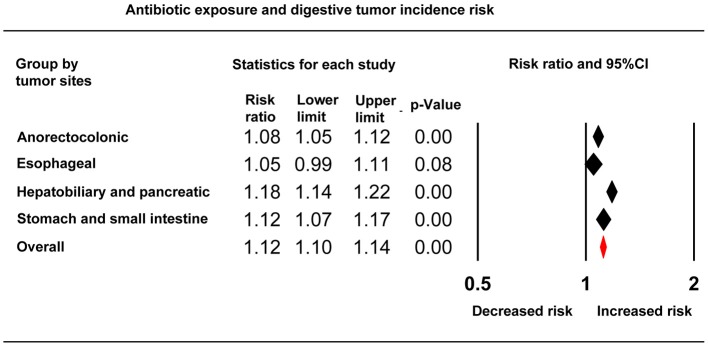
Forest plot showing the relationship between whole anti-bacterial drug exposure and digestive system cancer risks grouped by tumor site.

**Figure 3:**
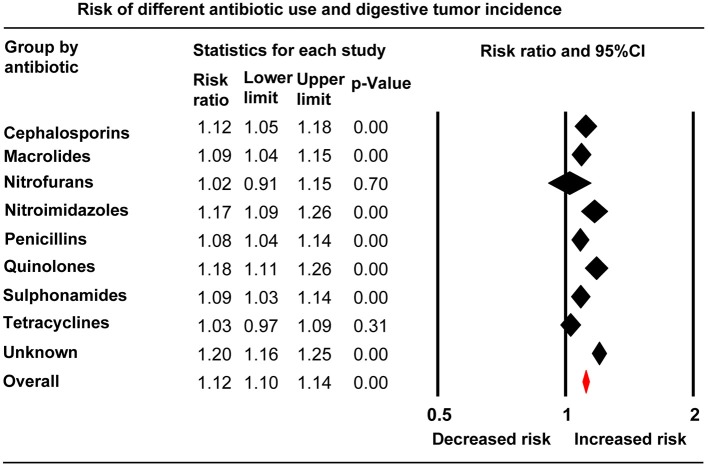
Forest plot of anti-bacterial drug use and risks of total digestive system cancers grouped by anti-bacterial drug class.

An increased risk of total digestive system cancers was associated with whole anti-bacterial drug exposure (RR, 1.12; 95% CI, 1.10–1.14). Slightly elevated cancer risks related to anti-bacterial drugs were also demonstrated in subgroups of stomach and small intestinal (RR, 1.12; 95% CI, 1.07–1.17), anorectocolonic (RR, 1.08; 95% CI, 1.05–1.12), hepatobiliary, and pancreatic cancers (RR, 1.18; 95% CI, 1.14–1.22). However, esophageal cancer (RR, 1.05; 95% CI, 0.99–1.10) showed no relationship to anti-bacterial drug use (Figure 2, Table 2). For the nine individual anti-bacterial drug groups, cephalosporins (RR, 1.12; 95% CI, 1.05–1.18), macrolides (RR, 1.09; 95% CI, 1.04–1.15), nitroimidazoles (RR, 1.17; 95% CI, 1.09–1.26), penicillins (RR, 1.08; 95% CI, 1.04–1.14), sulphonamides (RR, 1.09; 95% CI, 1.03–1.14), quinolones (RR, 1.18; 95% CI, 1.11–1.26), and the group of unknown anti-bacterial drugs (RR, 1.20; 95% CI, 1.16–1.25) statistically increased the risk of digestive cancers. However, nitrofurantoin and tetracycline showed no association with digestive tumor incidence (Figure 3, Table 2). We also calculated the RRs (95% CIs) to evaluate relationships between each anti-bacterial drug and the risk of cancer by organ site. Hepatobiliary, pancreatic, and anorectocolonic cancers were more closely related to anti-bacterial drug use, most notably with use of nitroimidazoles, sulphonamides, and quinolones (Table 2). The short-term use of anti-bacterial drugs (follow-up period < 5.98 years) showed no statistically significant association with cancer incidence (RR, 1.03; 95% CI, 0.99–1.07). Alternatively, the long-term use of anti-bacterial drugs slightly increased cancer risk, however, no obvious difference was seen between the 5.98–7.31 years group (RR, 1.15; 95% CI, 1.13–1.18) and the >7.31 years group (RR, 1.09; 95% CI, 1.01–1.18, Supplementary Figure 4).

**Table 2:** Risk assessment of each anti-bacterial drug class and digestive cancer incidence.

**Anti-bacterial drugs**	**Esophageal cancer**	**Stomach and small intestine cancer**	**Hepatobiliary and pancreatic cancer**	**Anorectocolonic cancer**	**Overall**
Cephalosporins	1.02 (0.81–1.28)	1.11 (0.90–1.37)	1.16 (1.05–1.29)	1.08 (0.98–1.19)	1.12 (1.05–1.18)
Macrolides	1.07 (0.88–1.31)	1.17 (0.97–1.41)	1.14 (1.03–1.25)	1.02 (0.95–1.10)	1.09 (1.04–1.15)
Nitrofurans	NA	1.89 (0.76–4.68)	0.96 (0.76–1.21)	1.04 (0.92–1.18)	1.02 (0.91–1.15)
Nitroimidazoles	1.08 (0.83–1.40)	1.11 (0.86–1.43)	1.22 (1.07–1.39)	1.21 (1.09–1.34)	1.17 (1.09–1.26)
Penicillins	1.06 (0.88–1.27)	1.05 (0.89–1.24)	1.11 (1.02–1.21)	1.07 (1.00–1.15)	1.08 (1.04–1.14)
Quinolones	1.13 (0.88–1.45)	1.13 (0.89–1.43)	1.26 (1.12–1.41)	1.16 (1.06–1.27)	1.18 (1.11–1.26)
Sulphonamides	0.94 (0.76–1.16)	1.05 (0.87–1.27)	1.13 (1.02–1.25)	1.11 (1.04–1.18)	1.09 (1.03–1.14)
Tetracyclines	1.02 (0.82–1.24)	1.05 (0.87–1.26)	1.09 (0.98–1.21)	0.98 (0.91–1.05)	1.03 (0.97–1.09)
Unknown	1.00 (0.81–1.24)	1.16 (1.06–1.27)	1.37 (1.27–1.48)	1.14 (1.07–1.21)	1.20 (1.16–.25)
Overall	1.05 (0.99–1.11)	1.12 (1.07–1.17)	1.18 (1.14–1.22)	1.08 (1.05–1.12)	1.12 (1.10–1.14)

### Anti-bacterial Drug and Gastric Cancer

Anti-bacterial drug exposure and gastric cancer event risk was evaluated in a combination of 11 studies, no obvious association was observed between gastric cancer and exposure to each individual anti-bacterial drug (Table 2). The RR (95% CIs) for antibacterial agents used to eradicate Hp was 1.11 (0.98–1.25), the risk was reduced to 0.69 (0.50–0.97) after excluding 262 patients with peptic ulcer disease (Supplementary Figures 2, 3).

### Drug Dose Accumulation and Digestive System Cancer Risk

**Figure 4:**
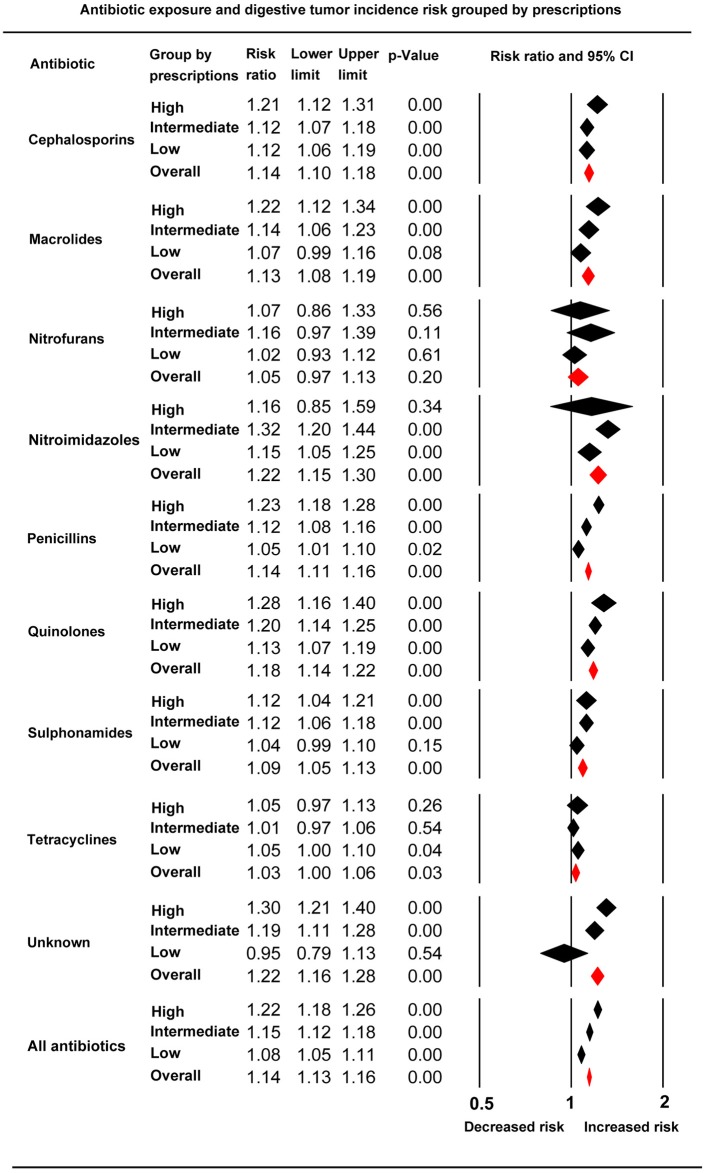
Forest plot of subgroup analysis based on anti-bacterial drug prescription accumulation in each individual anti-bacterial drug class and total anti-bacterial drugs.

Analysis of the subgroups of low, intermediate, and high anti-bacterial drug dose accumulation revealed that the digestive system cancer risks increased with the rise of medical prescriptions. The RRs (95% CIs) for the low, intermediate, and high groups were: 1.08 (95% CI, 1.05–1.11), 1.15 (95% CI, 1.12–1.18), and 1.22 (95% CI, 1.18–1.26), respectively. The dose accumulation effect on the risk of cancer was replicated in analyses among groups of cephalosporins, macrolides, penicillins, and quinolones (Figure 4).

## Discussion

We conducted the meta-analysis by combining 13 observational studies with four RCTs, a total of 77,284 digestive system cancer cases from a large population of 18,205,771 individuals were included in our analyses. Statistically significant associations between anti-bacterial drug exposure and the risk of digestive cancers occurrence were observed, especially in the subgroups of anorectocolonic, hepatobiliary, and pancreatic cancers. Although only modest associations were observed, a significant connection between higher drug dose accumulation and higher cancer risk further substantiated the correlations.

In our study, populations with exposures to nitroimidazoles and quinolones had a higher cancer risk than any other individual anti-bacterial drug class, these two anti-bacterial drug classes were commonly used to treat gastrointestinal infections or anaerobic bacterial infections (36, 37). Previous studies have suggested that recurrent infections and inflammatory diseases are related to higher cancer risks (38–40). Patients with weakened immune systems, such as smokers, diabetics, and those with cirrhosis, more frequently acquire infections requiring anti-bacterial drug treatment (41–44). Diabetes and cirrhosis were also identified as controlling factors that promote tumor development (42, 45). To our knowledge, three quarters of anti-bacterial drugs, such as cephalosporins, macrolides, sulphonamides, and penicillins, are used to treat respiratory tract infections (46). In the current study, there was no evidence to suggest a relationship between digestive system cancers and anti-bacterial drugs, however, slightly increased cancer risks were observed in our analysis. Thus, the correlation is unlikely to be induced completely by infection, and the anti-bacterial drugs are more likely used to treat infectious diseases related to digestive cancer, rather than a direct carcinogen.

A variety of studies support the theory that Helicobacter pylori (Hp) plays an important role in gastric cancer incidence and development (47, 48). Early Hp eradication is one important strategy in the prevention of gastric cancer (49). Anti-bacterial drugs used to treat Hp infections were correlated with a lower risk of gastric cancer in our analysis, and the risk was significantly reduced in patients without peptic ulcer diseases. Besides the effect of Hp eradication, there were two potential explanations for the decreased risks observed in our studies. First, the treatment course for Hp eradication is done over a short period of time (50), and increased cancer risk is generally observed in populations with long-term anti-bacterial drug exposure. Second, although Hp infection may be eradicated, irreversible initiation of tumorigenesis may exist in peptic ulcer patients.

There has been no evidence to suggest that anti-bacterial drugs have a direct carcinogenic effect on cancer development in previous investigations (51). We observed that high anti-bacterial drug dose accumulation and long-term anti-bacterial drug exposure significantly increased the risk of digestive cancer, which suggests that there is a relationship between anti-bacterial drug exposure and digestive system cancers. Our first hypothesis was that anti-bacterial drugs or their metabolites are carcinogens. Anti-bacterial drugs, including metronidazole and quinolones, have been proposed to have potential genotoxicity in previous studies (52, 53), and metronidazole has been shown to be metabolized to the carcinogen acetamide in the human body (54). Further, it has been suggested that anti-bacterial drugs are associated with colorectal cancer through increased the production of prostaglandins and up-regulation of cyclooxygenase-2, thus contributing to inflammation (55). Collectively, there is very limited evidence of a direct effect of anti-bacterial drugs on carcinogenesis.

Another possible explanation for the observed association is that anti-bacterial drug exposure induces changes in the composition of gut microbiota (56, 57). The important roles that vast microbial communities play in health and disease are being increasingly recognized. Disruption of the dynamic equilibrium disruption of the microbiota with the host can contribute to diseases, including malignancy (58). Previous studies have suggested that some microbes promote cancer development by regulating host cell signaling cascades or inducing histologic changes (59, 60). In addition, the intestinal microbiota has been associated with the inhibition of carcinogenesis due the important role these microbiota play in the conversion of health-related compounds, such as phytochemicals, into bioactive compounds (51, 61). Anti-bacterial drug exposure reduces the composition and diversity of the intestinal microbiota, predominately composed of anaerobes, causing loss of beneficial bacteria and an increase in pathogenic microbes (62, 63). These changes may elevate the risk of hepatic tissue or intestinal mucosa exposure to toxic bacterial metabolites or products that may be carcinogenic (57, 60). Further, the intestinal microbiota plays an essential role in maintaining immune balance (64), and anti-bacterial drug exposure may disturb the microbiota and reduce the immune barrier function of the microbiota, which protects against tumor development. Other potential biological mechanisms or underlying concerns between anti-bacterial drugs use and cancers were not clarified. The above explanations are consistent with our results. Of the anti-bacterial drugs commonly used to treat gastrointestinal infections, quinolones and nitroimidazoles were significantly associated with an increased risk of cancer. However, the less used tetracyclines showed no relationship to cancer events. These results should be considered by clinicians when prescribing these anti-bacterial drugs.

### Strengths and Limitations

To our knowledge, only one meta-analysis has evaluated the relationships between anti-bacterial drug exposure and cancer incidence risk. This study concluded that anti-bacterial drug ever-use is related to a slightly elevated breast cancer risk (65). A number of previous studies have investigated the uncertain association between anti-bacterial drugs and digestive system cancers. Our study was the first exhaustive meta-analysis conducted by combining these previous findings. We performed the analyses based on large study cohorts from multiple international population-based databases, including The Health Improvement Network database from the UK, the Population Register in Finland, the Swedish Inpatient Register, the Achmea Health Database in the Netherlands, and the Kaiser Permanente Medical Care Program in the USA. Each database provided comprehensive and representative data of medical diagnoses, demographics, and prescription pharmaceuticals prescribed for inpatients or outpatients. Finally, we conducted sub-analyses by drug dose accumulation and follow-up time, which further evaluated the association between anti-bacterial drugs and cancers.

Our study had several limitations. First, most of our included studies were observational, therefore, comprehensive information on residual confounding factors and the underlying diseases of patients was not available. If we ignore the dose accumulation response effect, the slightly increased risk of digestive cancer in our study may be partially explained by confounding factors and infections requiring anti-bacterial drug treatment. However, included studies were adjusted for some confounders such as sex, age, partial medical history including the use of acetylsalicylic acids, non-steroidal anti-inflammatory drugs and acetylsalicylic acids. Second, although we conducted subgroup analyses based on follow-up period, the follow-up time duration did not reflect the actual exposure dose of anti-bacterial drugs. Third, most of included studies were retrospective, although we have try our best to contact authors via email. The dose of anti-bacterial drug use was not obtained. Fourth, the included patients may receive multiple antibiotics at the same time. All of the data was retrieved from patient demographics database. We can only conducted subgroups analysis in different antibacterial drugs. Finally, lack of information on anti-bacterial drug treatment indications or pathogens is another notable limitation. If we combine anti-bacterial drug exposure and infectious pathogens, additional values in evaluating the relationship between anti-bacterial drug exposure and digestive system cancer incidence may exist.

In conclusion, anti-bacterial drug exposure slightly increased the risk of total digestive system cancer development, and the risks were elevated with the rise of anti-bacterial drug dose accumulation. Nitroimidazoles (mainly metroimidazole) and quinolones showed a closer relationship to cancer, especially in anorectocolonic tumors and hepatobiliary and pancreatic malignancies. Anti-bacterial drug exposure may be a treatment indicator rather than a direct carcinogen. Changes in intestinal microbiota or immune defense deregulation induced by anti-bacterial drugs may be the indirect cause of increased cancer risks. More comprehensive information about treatment indications or underlying diseases would be valuable information to combine with our analyses.

## Author Contributions

CB: study concept and design, acquisition of data, analysis and interpretation of data, statistical analysis, drafting of the manuscript, critical revision of the manuscript, and approved final submission. KW: study concept and design, analysis and interpretation of data, drafting of the manuscript, critical revision of the manuscript, approved final submission, and study supervision. YD: acquisition of data, analysis and interpretation of data, statistical analysis, drafting of the manuscript, and technical or material support. JK: study concept and design, analysis and interpretation of data, drafting of the manuscript, critical revision of the manuscript, and approved final submission.

### Conflict of Interest

The authors declare that the research was conducted in the absence of any commercial or financial relationships that could be construed as a potential conflict of interest.
