# Supplementary material for: Association Between Anti-bacterial Drug Use and Digestive System Neoplasms: A Systematic Review and Meta-analysis
Source: Front Oncol. 2019 Nov 27;9:1298. doi: 10.3389/fonc.2019.01298 (PMC6890852; doi:10.3389/fonc.2019.01298)
Supplement: Supplementary file 1 [file Data_Sheet_1.pdf]

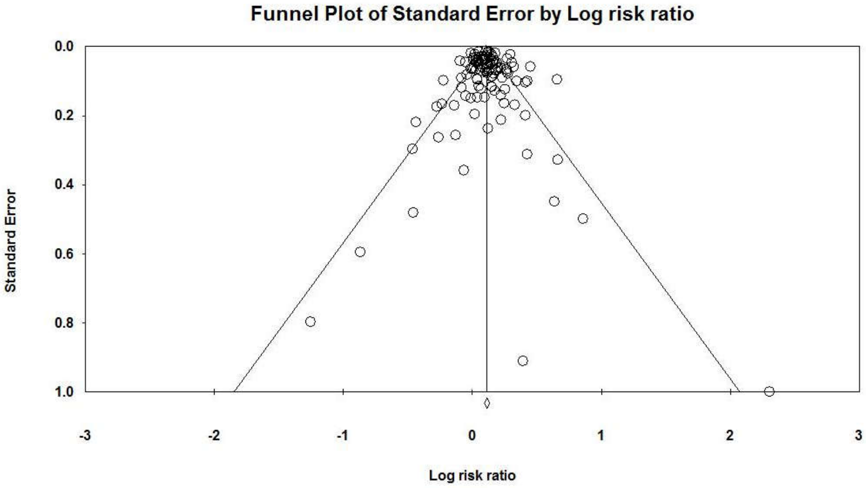

Supplementary Figure 1. The funnel plot did not show asymmetry and Egger's test (p = 0.68) indicated no obvious publication bias.

Antibiotic exposure and gastric cancer risk group by Hp eradication

Group by Hp eradication	study	Risk ratio	Lower limit	Upper limit	p-Value
No	Didham-2005-1	1.00	0.88	1.13	1.00
No	Didham-2005-2	1.19	0.93	1.53	0.17
No	Didham-2005-3	0.77	0.46	1.29	0.32
No	Didham-2005-4	1.10	0.82	1.47	0.52
No	Didham-2005-5	1.07	0.84	1.36	0.58
No	Didham-2005-6	1.89	0.78	4.56	0.16
No	Boursi-2015-1	1.18	1.09	1.28	0.00
No	Boursi-2015-2	1.09	1.03	1.15	0.00
No	Boursi-2015-3	1.03	0.95	1.12	0.48
No	Boursi-2015-4	1.03	0.94	1.14	0.48
No	Boursi-2015-5	1.13	1.03	1.25	0.01
No	Boursi-2015-6	1.11	0.97	1.28	0.13
No	Boursi-2015-7	1.16	1.08	1.24	0.00
No	Kilkkinen-2008-1	1.14	0.99	1.31	0.07
No	Kilkkinen-2008-2	1.28	0.93	1.77	0.13
No	Kilkkinen-2008-3	1.51	1.02	2.23	0.04
No	Kilkkinen-2008-4	1.10	1.02	1.19	0.02
No	Kilkkinen-2008-5	1.16	0.98	1.38	0.09
No	Kilkkinen-2008-6	1.51	1.23	1.85	0.00
No		1.12	1.07	1.18	0.00
Yes	Correa-2000	1.48	0.25	8.83	0.67
Yes	Li-2014	0.65	0.42	0.99	0.05
Yes	Zhou-2014	0.29	0.06	1.36	0.12
Yes	Lee-2013	0.94	0.46	1.89	0.86
Yes	Wong-2004	0.63	0.25	1.63	0.34
Yes	Wu-2009-1	1.05	0.96	1.14	0.27
Yes	Wu-2009-2	1.36	1.24	1.49	0.00
Yes	Take-2007	0.42	0.13	1.35	0.15
Yes		1.11	0.98	1.25	0.09
Overall		1.12	1.07	1.17	0.00

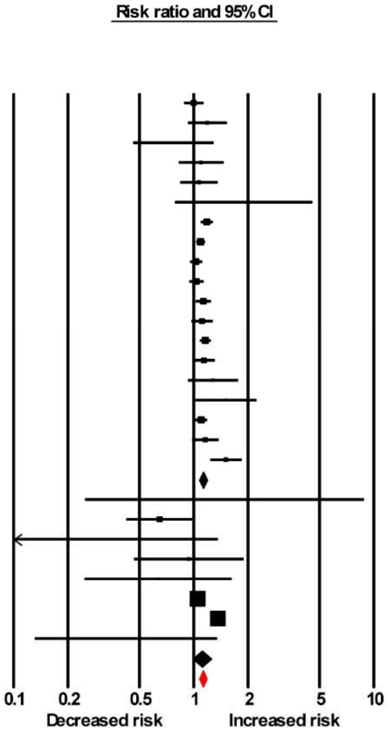

Supplementary Figure 2. The forest plot of antibiotic exposure and gastric cancer risk group by antibacterial drug used to treat Hp infection (or not).

Antibiotic exposure and gastric cancer risk group by Hp eradication excluded peptic ulcer population

Group by Hp eradication	study	Risk ratio	Lower limit	Upper limit	p-Value
No	Didham-2005-1	1.00	0.88	1.13	1.00
No	Didham-2005-2	1.19	0.93	1.53	0.17
No	Didham-2005-3	0.77	0.46	1.29	0.32
No	Didham-2005-4	1.10	0.82	1.47	0.52
No	Didham-2005-5	1.07	0.84	1.36	0.58
No	Didham-2005-6	1.89	0.78	4.56	0.16
No	Boursi-2015-1	1.18	1.09	1.28	0.00
No	Boursi-2015-2	1.09	1.03	1.15	0.00
No	Boursi-2015-3	1.03	0.95	1.12	0.48
No	Boursi-2015-4	1.03	0.94	1.14	0.48
No	Boursi-2015-5	1.13	1.03	1.25	0.01
No	Boursi-2015-6	1.11	0.97	1.28	0.13
No	Boursi-2015-7	1.16	1.08	1.24	0.00
No	Kilkkinen-2008-1	1.14	0.99	1.31	0.07
No	Kilkkinen-2008-2	1.28	0.93	1.77	0.13
No	Kilkkinen-2008-3	1.51	1.02	2.23	0.04
No	Kilkkinen-2008-4	1.10	1.02	1.19	0.02
No	Kilkkinen-2008-5	1.16	0.98	1.38	0.09
No	Kilkkinen-2008-6	1.51	1.23	1.85	0.00
No		1.12	1.08	1.16	0.00
Yes	Correa-2000	1.48	0.25	8.83	0.67
Yes	Li-2014	0.65	0.42	0.99	0.05
Yes	Zhou-2014	0.29	0.06	1.36	0.12
Yes	Lee-2013	0.94	0.46	1.89	0.86
Yes	Wong-2004	0.63	0.25	1.63	0.34
Yes		0.69	0.50	0.97	0.03
Overall		1.11	1.07	1.15	0.00

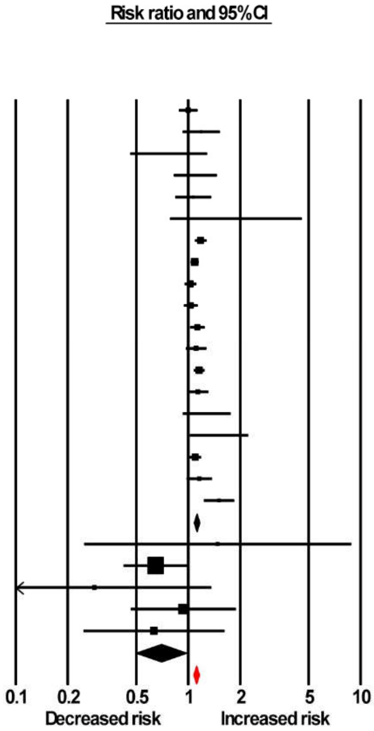

Supplementary Figure 3. The forest plot of antibacterial drug exposure and gastric cancer risk group by Hp eradication after excluding 262 cohorts with peptic ulcer disease. The RR reduced to 0.69 (95%CI, 0.50–0.97).

Antibiotic exposure and cancer risk group by follow-up period

Group by Follow-up period (years)	Statistics for each study: Risk ratio	Lower limit	Upper limit	p-Value
<5.98	1.03	0.99	1.07	0.16
5.98-7.31	1.15	1.13	1.18	0.00
7.31+	1.09	1.01	1.18	0.03
Overall	1.12	1.10	1.14	0.00

Risk ratio and 95% CI

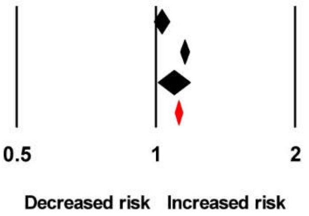

Supplementary Figure 4. The forest plot of antibacterial drug use and digestive system cancer risk group by follow-up period from the first antibacterial drug intake to first cancer diagnosis.

Supplementary Table 1. Number of patients exposure to different anti-bacterial drugs

First author, year	Drug types	Cancer types	Cancer patients	Exposure	Non-Exposure	Normal	Exposure	Non-Exposure
Yang, 2016(11)	All	Liver	1195	1013	182	4640	3712	928
Zhou, 2014(35)	All	Gastric	9	2	7	543	274	269
Vincent, 2016(23)	Penicillin	Colorectal	4029	1673	2356	15988	6293	9695
	Tetracyclines	Colorectal	4029	1158	2871	15988	4479	11509
	Sulfonamides and trimethoprim	Colorectal	4029	523	3506	15988	1903	14085
	Macrolides	Colorectal	4029	583	3446	15988	2292	13696
	Quinolones	Colorectal	4029	705	3324	15988	2524	13464
	Nitrofuran	Colorectal	4029	571	3458	15988	2153	13835
Boursi, 2015(10)	Penicillin	Esophagus	6108	2991	3117	23850	10569	13281
		Biliary	910	201	709	3543	649	2894
		Gallbladder	365	76	289	1426	282	1144
		Pancreas	4113	761	3352	16072	2682	13390
	Cephalosporin	Esophagus	6108	810	5298	23850	2847	21003
		Gastric	3859	569	3290	15022	1842	13180
		Hepatocellular	1299	200	1099	5055	577	4478
		Biliary	910	156	754	3543	537	3006
		Gallbladder	365	70	295	1426	233	1193
		Pancreas	4113	606	3507	16072	2186	13886
	Sulfacetamide	Esophagus	6108	836	5272	23850	3226	20624
		Gastric	3859	1833	2026	15022	6724	8298
		Gastric	3859	555	3304	15022	2094	12928
		Hepatocellular	1299	196	1103	5055	623	4432
		Biliary	910	178	732	3543	616	2927
		Gallbladder	365	70	295	1426	297	1129
		Pancreas	4113	729	3384	16072	2538	13534
	Tetracycline	Esophagus	6108	617	5491	23850	2247	21603
		Gastric	3859	384	3475	15022	1438	13584
		Hepatocellular	1299	141	1158	5055	456	4599
		Biliary	910	117	793	3543	382	3161
		Gallbladder	365	33	332	1426	167	1259
		Hepatocellular	1299	649	650	5055	2235	2820
		Pancreas	4113	423	3690	16072	1582	14490
	Quinolones	Esophagus	6108	496	5612	23850	1673	22177
		Gastric	3859	345	3514	15022	1164	13858

		Hepatocellular	1299	136	1163	5055	381	4674
		Biliary	910	103	807	3543	306	3237
		Gallbladder	365	43	322	1426	130	1296
		Pancreas	4113	384	3729	16072	1198	14874
	Nitroimidazoles	Esophagus	6108	232	5876	23850	828	23022
		Gastric	3859	163	3696	15022	557	14465
		Hepatocellular	1299	74	1225	5055	167	4888
		Biliary	910	483	427	3543	1755	1788
		Biliary	910	51	859	3543	145	3398
		Gallbladder	365	22	343	1426	84	1342
		Pancreas	4113	191	3922	16072	649	15423
		Gallbladder	365	184	181	1426	702	724
		Pancreas	4113	2011	2102	16072	7327	8745
	Macrolides	Esophagus	6108	1064	5044	23850	3806	20044
		Gastric	3859	714	3145	15022	2381	12641
		Hepatocellular	1299	248	1051	5055	762	4293
Take, 2007(29)	All	Gastric	13	9	4	1118	944	174
Lee, 2013(30)	All	Gastric	31	15	16	8211	4106	4105
Wang, 2014(28)	All	Colon	3593	3357	236	14372	13186	1186
	All	Rectal	1979	1795	184	7916	7125	791
Wong, 2004(34)	All	Gastric	18	7	11	1612	810	802
Correa, 2000(32)	All	Gastric	5	3	2	971	488	483
Li, 2014(33)	All	Gastric	84	33	51	2088	1053	1035
Boursi, 2015(27)	Penicillin	Colorectal	20990	11896	9094	82054	43776	38278
	Macrolides	Colorectal	20990	4393	16597	82054	16282	65772
	Cephalosporin	Colorectal	20990	3631	17359	82054	12480	69574
	Sulfacetamide	Colorectal	20990	4438	16552	82054	15154	66900
	Tetracycline	Colorectal	20990	2472	18518	82054	9756	72298
	Quinolones	Colorectal	20990	2339	18651	82054	7448	74606
	Nitroimidazoles	Colorectal	20990	1430	19560	82054	3885	78169

Supplementary Table 2. Newcastle-Ottawa Scale (NOS) for included 13 observational studies

First author, year	Representative of the exposed cohort	Selection of the nonexposed cohort	Ascertainment of exposure	Demonstration that outcome in interest was not present at start of the study	Comparability of cohorts on the basis of the design or analysis	Assessment of outcome	Was follow-up long enough for outcomes to occur?	Adequacy of follow-up of cohorts	Score
K Fall, 2006	★	★	★	★	★★	★	★		8
Kilkkinen, 2008	★	★	★	★	★	★	★		7
Take, 2007	★	★	★	★	★	★	★		7
Lee, 2013	★	★	★	★		★	★	★	7
Wu, 2009	★	★	★	★	★★	★	★		8
Didham, 2005	★	★	★	★	★	★	★	★	8
Friedman, 2009	★	★	★	★	★★	★	★	★	9
Jiun-Ling, 2014	★	★	★	★★	★	★	★	★	9
Friedman, 2009	★	★	★	★	★★	★	★	★	9
Boursi 2015	★	★	★	★	★	★	★	★	8
Boursi 2015	★	★	★	★	★	★	★	★	8
Vincent, 2016	★	★	★	★	★	★	★	★	8
Yang, 2016	★	★	★	★	★★	★	★	★	9

Supplementary Table 3. RCTs Quality evaluated by Cochrane Risk of Bias Tool

First author, year	Random sequence generation (selection bias)	Allocation concealment (selection bias)	Blinding of participants and personnel (performance bias)	Blinding of outcome assessment (attrition bias)	Selective reporting (reporting bias)	Other bias
Correa, 2000	■	■	□	□	■	■
Wong, 2004	■	■	■	■	■	■
Li, 2014	■	■	■	■	■	■
Zhou, 2014	■	○	■	○	■	■

□ high bias risk; ○ uncertain bias risk; ■ low bias risk.

Exact search strategy

PubMed

#1 (Anti-Bacterial Agents [Mesh Terms]) OR (Anti-Bacterial Compounds [Title/Abstract] OR Anti-Bacterial Compounds [Title/Abstract] OR Anti-infective agents [Title/Abstract] OR Antimycobacterial Agents Anti infective agents [Title/Abstract] OR Anti Mycobacterial Agents[Title/Abstract])

#2 Drug-Related Side Effects and Adverse Reactions [Mesh Terms]

#3 #1 OR #2

#4 (Neoplasms [Mesh Terms]) OR (Tumors[Title/Abstract] OR Tumor[Title/Abstract] OR Cancer[Title/Abstract] OR Cancers[Title/Abstract] OR Malignant Neoplasms[Title/Abstract] OR Neoplasm[Title/Abstract])

#5 (risk [Mesh Terms]) AND risk

#6 #3 AND #4 AND #5

Cochrane

#1 MeSH descriptor: [Anti-Bacterial Agents] explode all trees 12064

#2 Anti-Bacterial Compounds:ti,ab,kw or Anti-infective agents:ti,ab,kw or Anti Mycobacterial Agents:ti,ab,kw or Antimycobacterial Agents:ti,ab,kw (Word variations have been searched) 5843

#3 MeSH descriptor: [Neoplasms] explode all trees 78274

#4 Neoplasm:ti,ab,kw or "tumor":ti,ab,kw or "Cancer":ti,ab,kw or Benign Neoplasms:ti,ab,kw or Malignant Neoplasms (Word variations have been searched) 154759

#5 MeSH descriptor: [Risk] explode all trees 40143

#6 risk:ti,ab,kw (Word variations have been searched) 179725

#7 #1 or #2 16593

#8 #3 or #4 164550

#9 #5 or #6 182629

#10 #7 and #8 and #9

Embase

#8. #1 AND #6 AND #7

#7. #4 OR #5

#6. #2 OR #3

#5. ((('tumors'/exp/mj OR 'tumor'/exp/mj OR 'cancer'/exp/mj OR 'cancers'/exp/mj OR malignant) AND 'neoplasms'/exp/mj OR benign) AND 'neoplasms'/exp/mj OR 'neoplasm'/exp/mj) AND [english]/lim AND [humans]/lim AND [embase]/lim

#4. 'neoplasm'/exp/mj AND [english]/lim AND [humans]/lim AND [embase]/lim

#3. ((((('anti bacterial' AND compounds OR anti) AND bacterial AND compounds OR 'anti-infective') AND

agents OR anti) AND infective AND agents OR anti) AND mycobacterial AND agents OR antimycobacterial) AND agents AND [english]/lim AND [humans]/lim AND [embase]/lim

#2. 'antibiotic agent'/exp/mj AND [english]/lim AND [humans]/lim AND [embase]/lim

#1. 'risk'/exp/mj AND [english]/lim AND [humans]/lim AND [embase]/lim
